# Systemic inflammatory index correlates with cerebral small vessel disease in Parkinson’s disease

**DOI:** 10.3389/fnagi.2026.1748084

**Published:** 2026-06-03

**Authors:** Yongqing Cheng, Haiping Tang, Shuangfei You, Xin Wang, Lei Li, Songjie Chen, Shouru Xue, Guojun He

**Affiliations:** 1 Department of Neurology, The Yancheng Clinical College of Xuzhou Medical University, The First People’s Hospital of Yancheng, Yancheng, Jiangsu, China; 2 Department of Neurology, The First Affiliated Hospital of Soochow University, Suzhou, Jiangsu, China; 3 Department of Neurology, Binghai County People’s Hospital, Yancheng, Jiangsu, China; 4 Department of Radiology, The Yancheng Clinical College of Xuzhou Medical University, The First People’s Hospital of Yancheng, Yancheng, Jiangsu, China

**Keywords:** cerebral small vessel disease, inflammatory marker, Parkinson’s disease, systemic immune inflammation index, systemic inflammation response index

## Abstract

**Background:**

This study investigates the association between systemic inflammatory indices—specifically, the systemic immune inflammation index (SII) and systemic inflammation response index (SIRI)—and the severity of cerebral small vessel disease (CSVD) in patients with Parkinson’s disease (PD).

**Methods:**

A total of 167 patients with primary PD and complete MRI data were included. CSVD severity was assessed using total and modified CSVD burden scores, with patients categorized into two groups: mild (scores 0–1 or 0–2) and moderate–severe (scores 2–4 or 3–6). Logistic and ROC analyses were employed to evaluate the association and discriminative ability of SII and SIRI for CSVD severity.

**Results:**

Among 167 patients (59.3% male, mean age 69.6 ± 8.7 years), 91 and 96 were classified with moderate–severe CSVD based on total and modified scores, respectively. Higher SII and SIRI levels, along with a greater proportion of hypertension, were observed in the moderate–severe CSVD group. Spearman analysis indicated SII weakly correlated with both CSVD scores (total: *r* = 0.224, *p* = 0.004; modified: *r* = 0.155, *p* = 0.046), while SIRI showed a weak correlation with the total score only (*r* = 0.179, *p* = 0.021). After adjusting for covariates, logistic regression showed the highest tertiles of SII and SIRI were significantly associated with moderate–severe CSVD, whether using the total score (T3 vs. T1: OR 2.919, 95% CI 1.191–7.153 for SII; OR 4.295, 95% CI 1.681–10.973 for SIRI) or the modified score (T3 vs. T1: OR 4.196, 95% CI 1.705–10.327 for SII; OR 3.303, 95% CI 1.326–8.226 for SIRI).

**Conclusion:**

Although their individual discriminatory abilities are limited, elevated SII and SIRI are independently associated with more severe CSVD in PD patients.

## 1 Introduction

Parkinson’s disease (PD), the second most common neurodegenerative disease in the world, is predicted to have a prevalence of 267 per 100,000 population by 2050, a significant increase of 76% compared to 2021 (Su et al., 2025).

The pathogenesis of PD is multifactorial, with neuroinflammation recognized as a central contributor (Tansey et al., 2022). Microglial activation, reactive oxygen species production, and mitochondrial dysfunction within the substantia nigra accelerate the aggregation of *α*-synuclein, a pathological hallmark of PD (Forloni, 2023). Beyond the central nervous system, peripheral systemic inflammation also contributes to neurodegeneration. Elevated neutrophil counts, increased lymphocyte infiltration, and raised circulating pro-inflammatory cytokines have been documented both in the brains of PD patients and in animal models (Pajares et al., 2020). These observations suggest that peripheral immune dysregulation may communicate with the central nervous system, potentially through a compromised blood–brain barrier (BBB).

Cerebral small vessel disease (CSVD) represents a spectrum of pathological processes affecting the brain’s small arteries, arterioles, venules, and capillaries (Pantoni, 2010). CSVD is highly prevalent in older adults and is strongly associated with age-related neurological deficits, including gait impairment and cognitive decline (Wardlaw et al., 2019). Over the past decade, a growing body of evidence has documented an association between CSVD and PD. Cross-sectional studies have reported that PD patients with higher CSVD burden exhibit more severe motor dysfunction, particularly gait and postural instability (Chen et al., 2021; Ma et al., 2021). A recent meta-analysis further revealed that CSVD burden is associated with multi-domain cognitive impairment in PD (Wan et al., 2022). Importantly, longitudinal cohort studies have extended these findings by suggesting a potential temporal link: both the baseline severity of CSVD and its progression over time have been independently associated with the subsequent development of parkinsonism, supporting a potential contributory role of CSVD in PD neurodegeneration, though causality remains to be established (Jacob et al., 2023; Oveisgharan et al., 2021).

Inflammation has been implicated as a shared pathological mechanism that may bridge PD and CSVD. Chronic peripheral inflammation can induce endothelial dysfunction, promote BBB disruption, and facilitate the entry of peripheral immune cells into the perivascular space, thereby exacerbating both small vessel pathology and neurodegeneration (Low et al., 2019; Evans et al., 2021). In the specific context of PD, aggregated *α*-synuclein released from neurons can act as a damage-associated molecular pattern, activating peripheral monocytes and neutrophils via toll-like receptors and perpetuating a feed-forward loop of systemic inflammation (Tansey et al., 2022). This crosstalk between PD-related proteinopathy and systemic inflammation may accelerate CSVD progression in this patient population.

In recent years, composite systemic inflammatory indices that integrate information from multiple leukocyte subsets have attracted increasing attention. The systemic immune-inflammation index (SII; neutrophils × platelets/lymphocytes) and the systemic inflammation response index (SIRI; neutrophils × monocytes/lymphocytes) reflect both innate and adaptive immune activation more comprehensively than single leukocyte counts (Hu et al., 2014; Ma et al., 2023). Higher SII has been associated with an increased risk of PD in population-based studies and with worse motor function in PD patients (Liu et al., 2025; Li et al., 2021). In community-dwelling populations, elevated SII and SIRI have also been linked to greater CSVD burden (Xiao et al., 2023; Jiang et al., 2022). However, to date, no study has specifically examined whether these composite inflammatory markers correlate with CSVD severity within a PD cohort, which may harbor a distinct inflammatory milieu due to ongoing dopaminergic neurodegeneration.

Therefore, we conducted this cross-sectional study to investigate the associations between SII, SIRI, and the severity of CSVD in patients with PD. We hypothesized that higher levels of SII and SIRI would be independently associated with more severe CSVD burden, as assessed by two validated composite neuroimaging scores.

## 2 Materials and methods

This study recruited patients with PD who were hospitalized at the First People’s Hospital of Yancheng from June 2023 to October 2024. The study was approved by the Ethics Committee of the First People’s Hospital of Yancheng and was conducted in accordance with the principles of the Helsinki Declaration. Informed consent was obtained from all subjects.

### 2.1 Participants

The inclusion criteria are as follows: (1) age ≥ 18 years old, (2) primary PD that meets the diagnostic criteria for Movement Disorder Society (MDS), (3) able to provide a complete medical history and cooperate to complete the clinical scales and undergo cranial Magnetic Resonance Imaging (MRI) examination. The exclusion criteria include: (1) patients with atypical PD syndromes (such as progressive supranuclear palsy, multiple system atrophy, etc) or secondary Parkinson’s syndrome (such as vascular, drug-induced, etc.), (2) other diseases with MRI abnormalities, such ischemic stroke, cerebral hemorrhage, multiple sclerosis, brain trauma, brain tumors, etc. (3) combine other diseases that may affect systemic inflammation, such as infectious diseases, autoimmune diseases, hematological disorders, tumors, as well as severe functional disorders of the heart, liver and kidneys, (4) using antibiotics, glucocorticoids, immunosuppressants or other drugs that affect the systemic inflammatory state within 1 month, (5) incomplete image data.

### 2.2 Clinical and laboratory data

Upon admission, all the subjects’ baseline characteristics were collected through standard questionnaires, and the severity of PD was evaluated using clinical scales. Baseline characteristics included demographic information (gender, age, BMI), medical history (hypertension, diabetes, atrial fibrillation, coronary heart disease, and hyperlipidemia), the duration of PD, and the usage of related medications (evaluated using the Levodopa Equivalent Daily Dose (LEDD)). The clinical assessment of PD included the Unified Parkinson’s Disease Rating Scale (UPDRS) and Hoehn-Yahr (H&Y) stage, which were conducted by two trained and experienced neurologists.

All subjects had their venous blood drawn on the next morning of hospitalization after an overnight fast of more than 8 h. The blood samples were processed and the data were recorded by laboratory technicians who were blinded to the information of the subjects. The laboratory data included counts of white blood cells, neutrophils, lymphocytes, and platelets, lipid profile [triglycerides (TG), total cholesterol (TC), high-density lipoprotein cholesterol (HDL-C), low-density lipoprotein cholesterol (LDL-C), fasting plasma glucose (FPG), and glycosylated hemoglobin A1c (HbA1C)].

The systemic inflammation composite index, including SII and SIRI, was calculated using the following formulas: SII = neutrophils × platelets/lymphocytes; SIRI = neutrophils × monocytes/lymphocytes (Hu et al., 2014; Ma et al., 2023). LEDD was calculated according to the widely accepted conversion factors proposed by Tomlinson et al.: 100 mg levodopa = 100 mg levodopa controlled-release = 133 mg levodopa with entacapone = 1 mg pergolide = 1 mg pramipexole = 5 mg ropinirole = 10 mg bromocriptine = 100 mg amantadine = 10 mg selegiline oral = 1.25 mg selegiline sublingual = 100 mg safinamide (Tomlinson et al., 2010).

### 2.3 MRI acquisition and assessment

All participants underwent standardized brain MRI examination utilizing a 3.0 T scanner within 72 h of admission. The 3.0 T MRI protocol included the following parameters: axial T2-weighted (TR/TE 4000/100 ms, slice thickness 5 mm, gap 1 mm, matrix 512 × 512), axial fluid-attenuated inversion recovery (FLAIR) (TR/TE/TI 9000/120/2500 ms, slice thickness 5 mm), axial diffusion-weighted imaging (DWI) (TR/TE 5000/80 ms, b = 0 and 1,000 s/mm^2^, slice thickness 5 mm), 3D T1-weighted (TR/TE 7.2/3.1 ms, flip angle 9°, voxel size 1 × 1 × 1 mm^3^), and susceptibility-weighted imaging (SWI) (TR/TE 28/20 ms, slice thickness 2 mm).

Two experienced and well-trained radiologists, blinded to clinical information, independently analyzed the neuroimaging data. CSVD markers were identified using established criteria: white matter hyperintensities (WMH), including periventricular and deep WMH, were quantified through Fazekas scoring based on T2/FLAIR sequences. Cerebral microbleeds (CMBs) were defined as 2–10 mm hypointense foci on SWI. Lacunes appeared as 3–20 mm CSF-isointense cavities on T1, T2 or FLAIR sequences; Enlarged perivascular spaces (EPVS) manifested as <3 mm linear or ovoid CSF-signal spaces in basal ganglia (BG) or subcortical regions (Cannistraro et al., 2019; Wardlaw et al., 2013). Inter-rater reliability between the two radiologists was assessed using Cohen’s kappa (*κ*) for categorical CSVD markers (Fazekas periventricular and deep WMH grades, presence of lacunes, presence of CMB, and EPVS severity categories). The κ values ranged from 0.78 to 0.86, indicating substantial agreement. Any discrepancies were resolved by consensus discussion.

The CSVD burden was quantified using the original Staals scale and the modified Rothwell scale, both of which are validated composite scoring systems. The original Staals scale (0–4 points) allocated 1 point each for: ≥1 lacune; ≥1 CMB; Moderate–severe WMH (periventricular Fazekas 3 or deep Fazekas ≥2); Severe basal ganglia EPVS (≥11 counts).(Staals et al., 2014) The modified Rothwell scale (0–6 points) incorporated: 1 point for lacunes, moderate CMB burden (1–4), severe BG-EPVS (>20), or moderate WMH (grade 3–4); 2 points for extensive CMBs (≥5) or severe WMH (grade 5–6) (Lau et al., 2017). Based on these two scoring systems, the subjects were classified into mild CSVD (Staals 0–1; Rothwell 0–2) and moderate–severe CSVD (Staals 2–4; Rothwell 3–6) subgroups.

### 2.4 Statistics

Statistical analyses were performed by SPSS version 23.0 (IBM, New York, NY, USA). Continuous variables that follow a normal or non-normal distribution were described, respectively, by the mean ± standard deviation (SD) or by the median (25 and 75% interquartile). Categorical variables were presented as frequencies with percentages. All the subjects were divided into the mild CSVD subgroup and the moderate–severe CSVD subgroup based on the scores of the original Staals scale and the modified Rothwell scale. To better illustrate the characteristics, the subjects were also divided into three subgroups, based on the SII or SIRI tertiles. The comparison of baseline demographic and clinical characteristics between the mild CSVD and moderate–severe CSVD subgroups was analyzed using independent Student t-tests, one-way ANOVA, or Mann–Whitney U tests for continuous variables, and chi-square tests or Fisher’s exact tests for categorical variables. The Spearman rank correlation method was used to analyze the relationship between SII and SIRI and the total CSVD burden score (the Staals scale and the modified Rothwell scale). Univariable logistic regression analysis was conducted to screen the risk factors of moderate–severe CSVD. Subsequently, multivariate logistic regression analysis was conducted to determine the independent relationship between the levels of SII and SIRI and the severity of CSVD. We constructed two multivariate regression models to control for confounding factors: Model 1, adjusted for age and gender; Model 2, further adjusted for hypertension, diabetes, hyperlipidemia, coronary heart disease, smoking, drinking, BMI, TC, LDL and FPG. The neutrophil and lymphocyte were eliminated due to collinearity. Collinearity was assessed using variance inflation factors (VIF). Neutrophil count (VIF = 11.5) and lymphocyte count (VIF = 9.7) exceeded the conventional threshold of 5, confirming substantial collinearity with the composite indices SII and SIRI. These variables were therefore excluded from all multivariable models. An association was indicated as the odds ratio (OR) or adjusted odds ratio (aOR) with the 95% confidence interval (CI). Restrictive cubic splines (RCS) were used to assess the possible nonlinear correlations and dose–response correlations between SII and SIRI and moderate–severe CSVD. For restricted cubic spline analyses, three knots were positioned at the 10th, 50th, and 90th percentiles of the SII and SIRI distributions, following the recommendations of Harrell (2015). The median value of each index served as the reference. The ROC curve was further performed to assess the predictive value of SII or SIRI for moderate–severe CSVD. Statistical significance was defined as a two-sided *p*-value of < 0.05.

## 3 Results

### 3.1 Baseline characteristics

**Figure 1:**
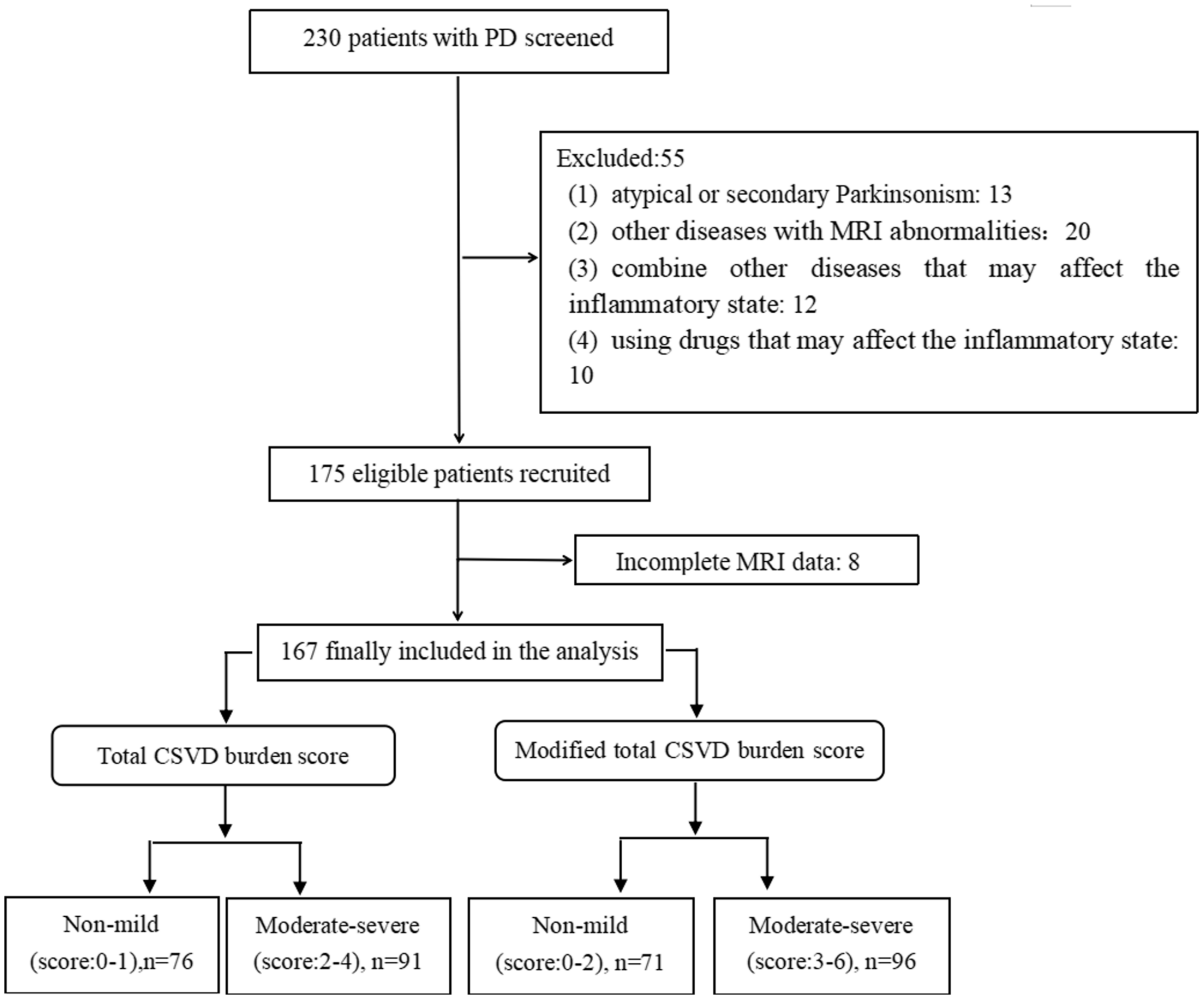
Flowchart of the study.

From May 2023 to October 2024, A total of 230 patients with PD were consecutively screened in this study. 63 patients were excluded for various reasons according to the exclusion criteria (Figure 1). Ultimately, 167 participants were recruited for the study. The average age was 69.6 ± 8.7 years, with 99 (59.3%) being male. The median and interquartile range (IQR) of SII was 428.00 (243.68, 665.60), and that of SIRI was 0.968 (0.674, 1.600). The SII and SIRI levels of all subjects were categorized into tertiles, defined as follows: Tertile 1 (SII ≤ 302.29; SIRI ≤ 0.76), Tertile 2 (SII = 302.30–509.22; SIRI = 0.77–1.25), and Tertile 3 (SII ≥ 509.23; SIRI ≥ 1.26). For the SII tertile subgroups (T1-T3), the total CSVD burden scores (based on the Staals scale) were 1 (1, 3), 2 (1, 3), and 3 (1, 3), respectively, showing statistically significant differences among the three groups (*p* = 0.007). The modified total CSVD burden scores (based on the Rothwell scale) were 2 (2, 4), 3 (2, 4), and 3 (2, 4), respectively, also demonstrating a statistically significant difference (*p* = 0.028). Regarding the SIRI tertile subgroups (T1-T3), the total CSVD burden scores were 1 (1, 3), 1 (1, 3), and 3 (1, 3), respectively, with significant differences (*p* = 0.013). However, the modified total CSVD burden scores were 2 (2, 4), 3 (2, 4), and 3 (2, 4), without showing statistically significant differences among the groups (*p* = 0.224).

### 3.2 Comparison of characteristics between patients with different severities of CSVD

All patients were divided into two subgroups based on the severity of CSVD: the no-mild CSVD subgroup (the total CSVD burden score 0–1 or modified total CSVD burden score 0–2) and the moderate–severe CSVD subgroup (the total CSVD burden score 2–4 or modified total CSVD burden score 3–6). The comparison of characteristics between the two subgroups was presented in Table 1. Compared with the mild CSVD subgroup, patients in the moderate–severe CSVD subgroup had higher levels of SII and SIRI, and a higher proportion of hypertension (all *p* < 0.05). Notably, total white blood cell counts did not differ significantly between the two groups, whereas the differential counts showed significant or near-significant differences. This supports the concept that composite indices such as SII and SIRI, which integrate information from multiple leukocyte subsets, are more sensitive to the underlying inflammatory imbalance than total leukocyte counts alone. Furthermore, when using the total CSVD burden (Staals scale) for grouping, patients in the moderate-to-severe CSVD subgroup also had higher levels of age and neutrophil counts. However, when using the modified CSVD burden score (Rothwell scale) for grouping, patients in the moderate-to-severe CSVD subgroup had higher levels of lymphocyte counts and a higher proportion of hyperlipidemia. There were no significant statistical differences in the other variables between the two subgroups.

**Table 1:** Demographic and clinical characteristics according to the severity of CSVD.

Characteristics	Total CSVD burden score		*p* value	Modified total CSVD burden score		*p* value
	Mild (*n* = 76)	Moderate-severe (*n* = 91)		Mild (*n* = 71)	Moderate–severe (*n* = 96)	
Demographics						
Male, *n* (%)	40 (52.6)	59 (64.8)	0.110	40 (56.3)	59 (61.5)	0.506
Age, mean(SD) (years)	66.9 (8.7)	71.8 (8.1)	<0.001	68.1 (9.0)	70.7 (8.4)	0.057
BMI, mean (SD)(Kg/cm^2^)	24.06 (2.74)	24.03 (2.84)	0.947	24.05 (3.07)	24.03 (2.57)	0.966
Medical history, *n* (%)						
Hypertension	23 (30.3)	43 (47.3)	0.025	20 (28.2)	46 (47.9)	0.010
Diabetes mellitus	11 (14.5)	17 (18.7)	0.469	8 (11.3)	20 (20.8)	0.102
Coronary artery disease	10 (13.2)	10 (11.0)	0.667	8 (11.3)	12 (12.5)	0.808
Atrial fibrillation	4 (5.3)	3 (3.3)	0.528	5 (7.0)	2 (2.1)	0.114
Hyperlipidemia	7 (9.2)	4 (4.4)	0.212	9 (12.7)	2 (2.1)	0.006
Smoking	20 (26.3)	30 (33.0)	0.350	20 (28.2)	30 (31.3)	0.667
Drinking	17 (22.4)	29 (31.9)	0.171	17 (23.9)	29 (30.2)	0.370
Clinical characteristics, median (IQR)						
Disease duration	2 (1, 5)	3 (1, 6)	0.928	2.3 (1, 5)	2 (1, 5)	0.584
LEDD	425 (375.00, 493.75)	425 (375, 575)	0.367	425 (375, 500)	425 (375, 571.55)	0.387
H&Y stage	2.5 (2, 3)	2.5 (1.5, 3)	0.784	2 (2, 3)	2.5 (1.63, 3)	0.550
UPDRS I	3.5 (2, 6)	5 (2, 8)	0.453	6 (3, 10.8)	8 (5.10)	0.112
UPDRS II	12 (8, 19.8)	14 (8, 22)	0.239	12 (6, 20)	14 (9.21.8)	0.130
UPDRS III	27 (22, 45)	33 (20, 48)	0.474	27 (21, 45)	33.5 (20, 47)	0.267
Laboratory characteristics, median (IQR)						
White blood cell count (× 10^9^/L)	5.85 (4.53, 8.00)	6.47 (4.83, 8.52)	0.148	6.00 (4.62, 8.23)	6.05 (4.67, 8.22)	0.603
Neutrophil count (× 10^9^/L)	3.21 (2.43, 4.84)	3.91 (2.94, 5.90)	0.035	3.27 (2.44, 5.10)	3.86 (2.86, 5.20)	0.109
Lymphocyte count (× 10^9^/L)	1.70 (1.33, 2.20)	1.50 (1.20, 2.00)	0.119	1.70 (1.40, 2.20)	1.50 (1.19, 1.94)	0.021
Monocyte count (× 10^9^/L)	0.45 (0.32, 0.56)	0.44 (0.32, 0.60)	0.530	0.46 (0.30, 0.58)	0.44 (0.35, 0.59)	0.564
Platelet count (× 10^9^/L)	195 (140.75, 236.25)	195 (155, 241)	0.368	185 (143, 232)	197.5 (156, 241)	0.308
FPG (mmol/L)	5.12 (4.55, 5.79)	5.35 (4.56, 6.30)	0.197	5.11 (4.50, 5.76)	5.31 (4.56, 6.39)	0.179
TC (mmol/L)	4.38 (1.22)	4.39 (1.19)	0.953	4.43 (1.32)	4.35 (1.11)	0.660
TG (mmol/L)	1.21 (1.00, 1.69)	1.19 (0.92, 1.76)	0.684	1.21 (0.95, 1.80)	1.19 (0.97, 1.68)	0.761
LDL-C (mmol/L)	2.61 (0.78)	2.70 (0.82)	0.481	2.69 (0.81)	2.63 (0.80)	0.643
Uric acid	306.20 (83.51)	329.03 (93.12)	0.100	305.81 (85.11)	328.13 (91.63)	0.111
SII	374.50 (214.39, 498.07)	462.28 (302.29, 841.46)	0.005	321.02 (207.90, 509.22)	456.28 (344.43, 760.38)	0.003
SIRI	0.849 (0.588, 1.186)	1.098 (0.754, 2, 011)	0.008	0.869 (0.510, 1.209)	1.063 (0.748, 1.787)	0.010
SII, *n* (%)			0.012			0.001
T1	32 (42.1)	23 (25.3)		35 (49.3)	20 (20.8)	
T2	27 (35.5)	29 (31.9)		19 (26.8)	37 (38.5)	
T3	17 (22.4)	39 (42.9)		17 (23.9)	39 (40.6)	
SIRI, *n* (%)			0.002			0.024
T1	32 (42.1)	24 (26.4)		30 (42.3)	26 (27.1)	
T2	29 (38.2)	26 (28.6)		25 (35.2)	30 (31.3)	
T3	15 (19.7)	41 (45.1)		16 (22.5)	40 (41.7)	

CSVD, cerebral small vessel disease; SD, standard deviation; BMI, body mass index; LEDD, Levodopa equivalent daily dose; H&Y, Hoehn-Yahr; UPDRS, Unified Parkinson’s Disease Rating Scale; IQR, interquartile range; FPG, fasting plasma glucose; TC, total cholesterol; TG, triglyceride; LDL-C, low-density lipoprotein cholesterol; SII, Systemic immune-inflammation index; SIRI, systemic inflammation response index.

### 3.3 Correlation between SII, SIRI, and the CSVD-related scores

**Figure 2:**
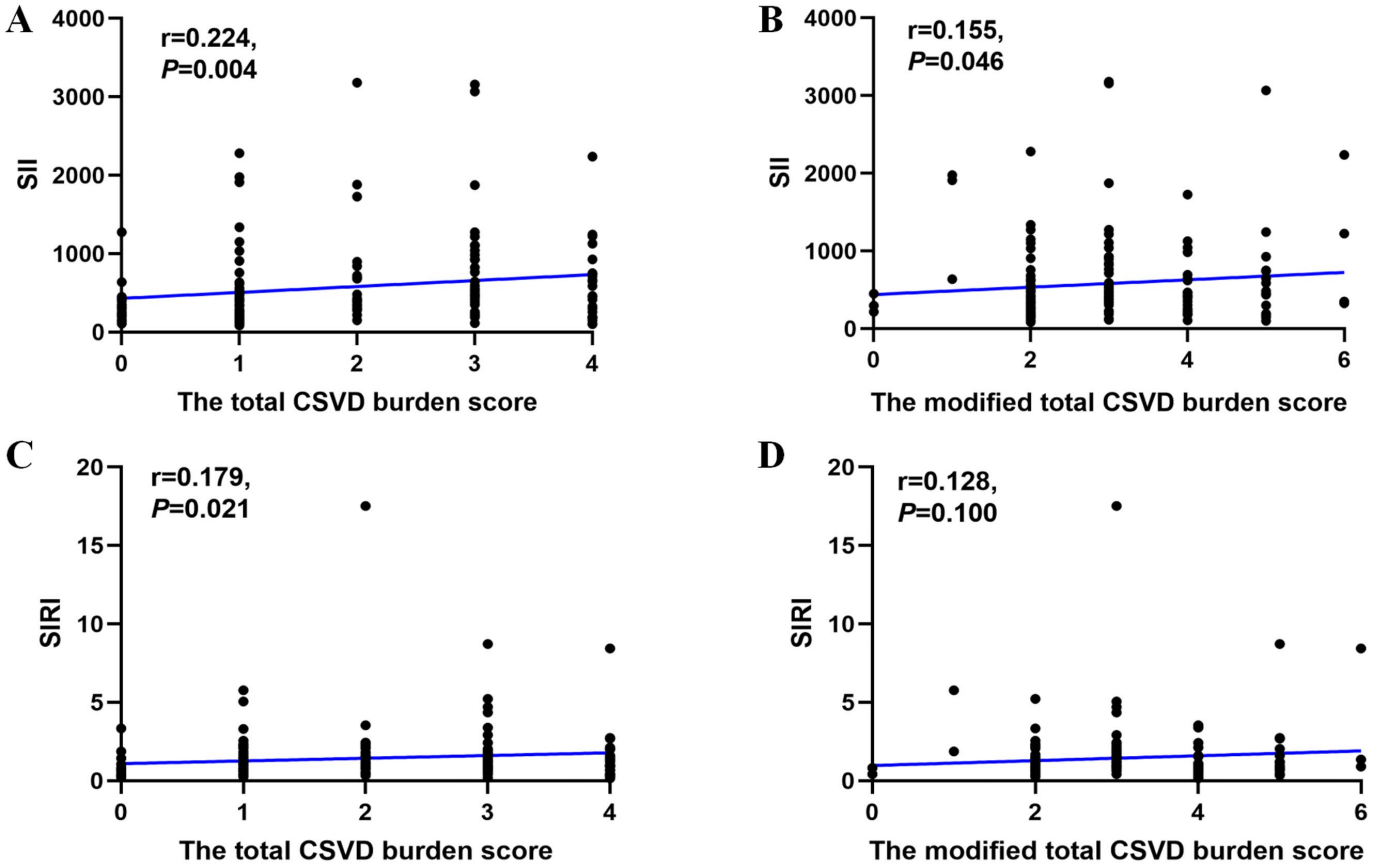
Spearman correlation analysis between SII/SIRI and the severity of CSVD. **(A)** Spearman correlation analysis between SII and the total CSVD burden score. **(B)** Spearman correlation analysis between SII and the modified total CSVD burden score. **(C)** Spearman correlation analysis between SIRI and the total CSVD burden score. **(D)** Spearman correlation analysis between SIRI and the modified total CSVD burden score.

Spearman’s correlation analysis was used to explore the correlation between SII, SIRI and the total CSVD, and modified total CSVD burden scores. The results showed that SII and SIRI were significantly correlated with the total CSVD burden score (*r* = 0.224, *p* = 0.004 and *r* = 0.179, *p* = 0.021, respectively), but only SII, not SIRI, was correlated with the modified total CSVD burden score (*r* = 0.155, *p* = 0.046 and *r* = 0.128, *p* = 0.100, respectively) (Figure 2). The subsequent analysis using restricted cubic splines (RCS) will further examine the possibility of nonlinearity.

### 3.4 Associations of SII and SIRI with the severity of CSVD

The results of univariate and multivariate logistic regression analyses for moderate–severe CSVD using SII and SIRI as continuous variables and tertiles were presented in Tables 2, 3. The results of univariate logistic regression analysis indicate that when using the CSVD total burden score as the grouping criterion, age, hypertension, neutrophil, SII, and SIRI are risk factors for moderate–severe CSVD, regardless of whether SII and SIRI were treated as continuous variables or tertile variables. When using the modified CSVD total burden score as the grouping criterion, hypertension, hyperlipidemia, lymphocyte, SII, and SIRI are potential risk factors for moderate–severe CSVD. It should be noted that SIRI is only a potential risk factor when it is treated as a tertile variable rather than a continuous variable.

**Table 2:** Univariate analyses for the potential risk factors associated with moderate–severe CSVD.

Baseline characteristics	Total CSVD burden score		Modified total CSVD burden score	
	OR (95%CI)	*p* value	OR (95%CI)	*p* value
Demographics				
Male, *n* (%)	0.603 (0.323–1.123)	0.111	0.809 (0.434–1.510)	0.506
Age, mean(SD) (years)	1.072 (1.031–1.114)	<0.001	1,035 (0.999–1.073)	0.059
BMI, mean (SD)(Kg/cm^2^)	0.996 (0.893–1.112)	0.947	0.998 (0.893–1.114)	0.966
Medical history, *n* (%)				
Hypertension	2.064 (1.089–3.913)	0.026	2.346 (1.220–4.512)	0.011
Diabetes mellitus	1.357 (0.593–3.108)	0.470	2.072 (0.855–5.023)	0.107
Coronary artery disease	0.815 (0.320–2.075)	0.668	1.125 (0.434–2.916)	0.808
Atrial fibrillation	0.614 (0.133–2.831)	0.531	0.281 (0.053,1.492)	0.136
Hyperlipidemia	0.453 (0.127–1.611)	0.221	0.147 (0.031,0.701)	0.016
Smoking	1.377 (0.703–2.697)	0.351	1.159 (0.591–2.273)	0.667
Drinking	1.623 (0.809–3.258)	0.173	1.375 (0.684–2.763)	0.371
Clinical characteristics				
Disease duration	1.020 (0.932–1.115)	0.670	0.996 (0.911–1.090)	0.933
LEDD	1.001 (0.999–1.002)	0.429	1.001 (0.999–1.003)	0.362
H&Y stage	1.068 (0.793–1.437)	0.666	1.042 (0.773–1.405)	0.787
UPDRS I	1.019 (0.949–1.095)	0.599	1.021 (0.950–1.098)	0.570
UPDRS II	1.019 (0.986–1.053)	0.270	1.022 (0.988–1.057)	0.213
UPDRS III	1.007 (0.990–1.025)	0.415	1.008 (0.991–1.026)	0.354
Laboratory characteristics				
White blood cell count (× 10^9^/L)	1.110 (0.980–1.258)	0.101	1.047 (0.926–1.184)	0.466
Neutrophil count (× 10^9^/L)	1.196 (1.022–1.400)	0.026	1.135 (0.973–1.324)	0.106
Lymphocyte count (× 10^9^/L)	0.719 (0.466–1.109)	0.135	0.592 (0.375–0.935)	0.025
Monocyte count (× 10^9^/L)	2.064 (0.357–11.947)	0.418	1.192 (0.206–6.895)	0.844
Platelet count (× 10^9^/L)	1.002 (0.998–1.007)	0.334	1.002 (0.998–1.007)	0.380
FPG (mmol/L)	1.092 (0.941–1.268)	0.247	1.065 (0.919–1.235)	0.414
TC (mmol/L)	1.008 (0.781–1.299)	0.953	0.944 (0.730–1.219)	0.658
TG (mmol/L)	1.057 (0.782–1.427)	0.719	0.995 (0.739–1.339)	0.974
LDL-C (mmol/L)	1.149 (0.783–1.685)	0.479	0.913 (0.622–1.340)	0.641
Uric acid	1.003 (0.999–1.007)	0.102	1.003 (0.999–1.006)	0.112
SII	1.001 (1.000–1.001)	0.020	1.001 (1.000–1.001)	0.037
SIRI	1.402 (1.021–1.925)	0.037	1.333 (0.982–1.810)	0.065
SII, *n* (%)				
T1	Reference			
T2	1.494 (0.706–3.162)	0.294	3.408 (1.563–7.432)	0.002
T3	3.192 (1.460–6.977)	0.004	4.015 (1.820–8.858)	0.001
SIRI, *n* (%)				
T1	Reference			
T2	1.195 (0.565–2.527)	0.640	1.385 (0.656–2.921)	0.393
T3	3.644 (1.648–8.060)	0.001	2.885 (1.319–6.307)	0.008

CSVD, cerebral small vessel disease; SD, standard deviation; BMI, body mass index; LEDD, Levodopa equivalent daily dose; H&Y, Hoehn-Yahr; UPDRS, Unified Parkinson’s Disease Rating Scale; IQR, interquartile range; FPG, fasting plasma glucose; TC, total cholesterol; TG, triglyceride; LDL-C, low-density lipoprotein cholesterol; SII, Systemic immune-inflammation index; SIRI, systemic inflammation response index; OR, odds ratio; CI, confidence interval.

**Table 3:** Multivariate regression analyses for moderate–severe CSVD with FAR as continuous variables.

Characteristics	Model 1		Model 2	
	aOR (95% CI)	*p* value	aOR (95% CI)	*p* value
CSVD burden score				
SII				
Tertile 1	Reference		Reference	
Tertile 2	1.296 (0.593, 2.830)	0.516	1.215 (0.528, 2.798)	0.647
Tertile 3	3.017 (1.323, 6.879)	0.009	2.919 (1.191, 7.153)	0.019
As continuous variable	1.001 (1.000, 1.002)	0.058	1.001 (1.000–1.001)	0.099
SIRI				
Tertile 1	Reference		Reference	
Tertile 2	1.176 (0.536, 2.577)	0.686	1.169 (0.513, 2.668)	0.710
Tertile 3	3.380 (1.458, 7.834)	0.005	4.295 (1.681, 10.973)	0.002
As continuous variable	1.336 (0.968, 1.845)	0.078	1.362 (0.953, 1.946)	0.090
Modified CSVD burden score				
SII				
Tertile 1	Reference		Reference	
Tertile 2	3.238 (1.474–7.111)	0.003	3.221 (1.347–7.704)	0.009
Tertile 3	3.922 (1.751–8.788)	0.001	4.196 (1.705–10.327)	0.002
As continuous variable	1.001 (1.000–1.001)	0.059	1.001 (1.000–1.002)	0.073
SIRI				
Tertile 1	Reference		Reference	
Tertile 2	1.410 (0.659–3.017)	0.377	1.441 (0.632–3.289)	0.385
Tertile 3	2.817 (1.252–6.334)	0.012	3.303 (1.326–8.226)	0.010
As continuous variable	1.309 (0.959–1.785)	0.090	1.303 (0.923–1.839)	0.132

The neutrophil and lymphocyte were not included in the multivariable regression analysis because of collinearity. Model 1: adjusted for gender and age, Model 2: further adjusted for hypertension, diabetes, hyperlipidemia, coronary heart disease, smoking, drinking, BMI, TC, LDL, FPG based on Model 1. CSVD, cerebral small vessel disease; SII, Systemic immune-inflammation index; SIRI, systemic inflammation response index; aOR, adjusted odds ratio; CI, confidence interval.

Subsequently, we established two multivariate regression models for multivariate regression analysis. The results showed that after adjusting for all covariates, when SII and SIRI were treated as continuous variables, their correlation with moderate–severe CSVD was not significant. However, when SII and SIRI were treated as tertiles, both high levels of SII and SIRI were significantly associated with the occurrence of moderate–severe CSVD, regardless of whether the total CSVD or the modified total CSVD burden score was used as the grouping criterion.

When the tertile 1 was used as the reference, the adjusted odds ratio (aOR) for SII T3 versus T1 was 2.919 (95% CI 1.191–7.153) based on the total CSVD burden score (Staals scale) and 4.196 (95% CI 1.705–10.327) based on the modified score (Rothwell scale). For SIRI, the aOR for T3 versus T1 was 4.295 (95% CI 1.681–10.973) and 3.303 (95% CI 1.326–8.226), respectively. Furthermore, in the fully adjusted model (Model 2), we made additional adjustments to LEDD for a sensitivity analysis, and the results remained essentially unchanged (Supplementary Table 1).

### 3.5 The dose–response correlation of SII and SIRI with moderate to severe CSVD

**Figure 3:**
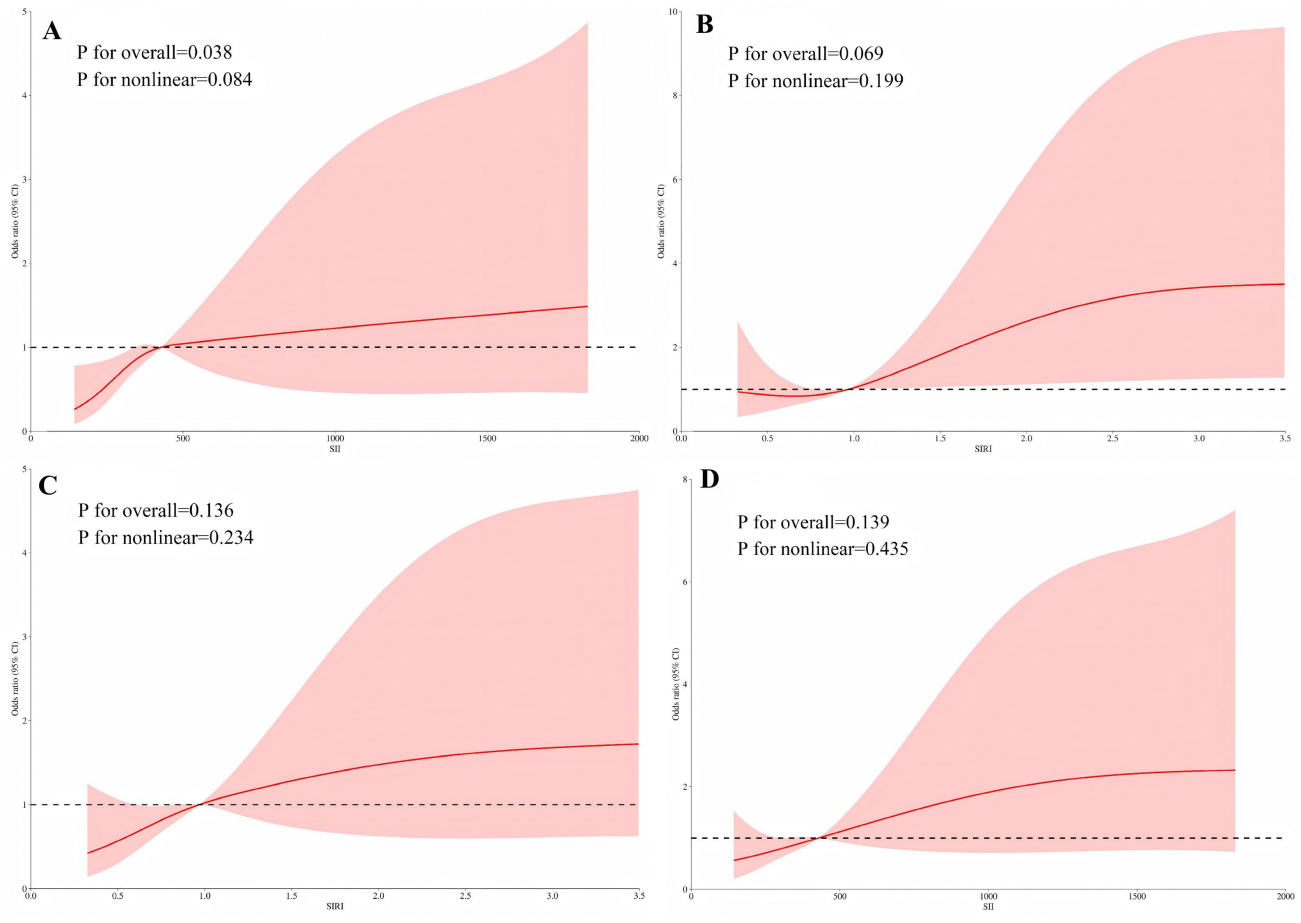
The dose–response correlation of SII and SIRI with moderate to severe CSVD. **(A)** The restricted cubic spline (RCS) for nonlinear correlations and dose–response correlations between SII and the total CSVD burden score. **(B)** The restricted cubic spline (RCS) for nonlinear correlations and dose–response correlations between SII and the modified total CSVD burden score. **(C)** The restricted cubic spline (RCS) for nonlinear correlations and dose–response correlations between SIRI and the total CSVD burden score. **(D)** The restricted cubic spline (RCS) for nonlinear correlations and dose–response correlations between SIRI and the modified total CSVD burden score.

To explore the possibility of nonlinearity between SII and SIRI and moderate-to-severe CSVD, as well as their dose–response relationship, we further conducted restricted cubic spline (RCS) analysis. The results showed that after adjusting for all the covariates, neither SII nor SIRI exhibited non-linear correlations with moderate-to-severe CSVD, regardless of using the Rothwell scale or the Rothwell scale as the grouping criterion (Figure 3).

### 3.6 ROC analysis of the predictive value of SII and SIRI for moderate–severe CSVD

**Figure 4:**
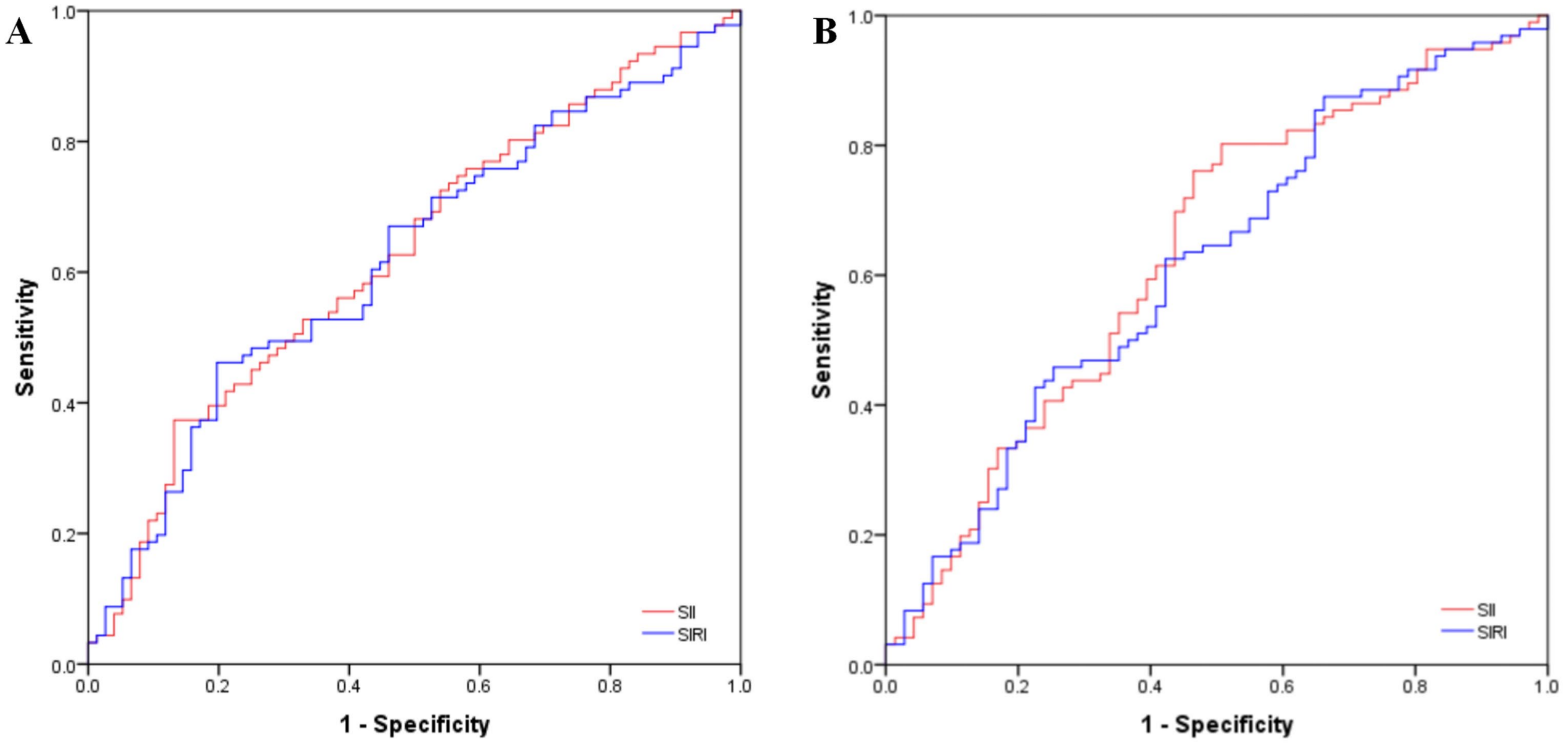
The ROC analysis of the predictive value of SII and SIRI for moderate–severe CSVD. **(A)** The ROC analysis of the predictive value of SII and SIRI for moderate–severe CSVD based on the total CSVD burden score. **(B)** The ROC analysis of the predictive value of SII and SIRI for moderate–severe CSVD based on the modified total CSVD burden score.

The results of the ROC analysis are presented in (Figure 4). The results indicated that when the Staals scale was used as the grouping criterion, the AUC of SII and SIRI for predicting moderate–severe CSVD were 0.627 (95% CI = 0.542–0.711, *p* = 0.005) and 0.620 (95% CI = 0.535–0.705, *p* = 0.008), respectively. When the Rothwell scale was used as the grouping criterion, the AUC of SII and SIRI for predicting moderate–severe CSVD were 0.635 (95% CI = 0.549–0.722, *p* = 0.003) and 0.615 (95% CI = 0.530–0.702, *p* = 0.010). In exploratory analyses, we constructed composite models by combining SII or SIRI with age and hypertension. When age and hypertension were added to SII, the AUC increased to 0.698 (95% CI 0.619–0.777) for the Staals scale and 0.746 (95% CI 0.671–0.821) for the Rothwell scale. Similar improvements were observed when SIRI was combined with age and hypertension (AUC 0.684 and 0.664, respectively). Detailed results are provided in Supplementary Table 2.

## 4 Discussion

The present study demonstrates that elevated peripheral systemic inflammatory indices (SII and SIRI) are significantly associated with the severity of CSVD in PD patients. The levels of SII and SIRI in patients with moderate–severe CSVD were significantly higher compared to those with mild CSVD. Meanwhile, compared with the first tertile, patients in the highest tertile of SII or SIRI had a significantly increased risk of combined moderate-to-severe CSVD, regardless of which scale was used as the grouping criterion. While earlier epidemiological studies established associations between systemic inflammation and PD progression (Zhao et al., 2025), our results extend these observations by quantitatively correlating composite inflammatory indices with neuroimaging biomarkers of CSVD severity, focusing on the potential cerebral vascular pathology of PD. Interestingly, in the Spearman correlation analysis, the correlation between SIRI and the CSVD score varied depending on the different assessment scales used, suggesting that different CSVD assessment methods might have an impact on this correlation. The discrepancy between Spearman correlation and logistic regression results for SIRI, depending on the CSVD scale used, warrants careful interpretation. Spearman correlation evaluates linear monotonic trends with continuous scores, whereas tertile-based logistic regression captures non-linear threshold effects and group separation. To further explore this discrepancy, we examined the distribution of CSVD components according to each scale. The Rothwell scale classified more patients with moderate cerebral microbleed burden (1–4 CMB) and moderate-to-severe white matter hyperintensities (grade 3–4) as ‘moderate–severe’ compared with the stricter Staals scale (which requires severe BG-EPVS ≥11 for EPVS points and treats moderate CMB equally as ≥1). This redistribution may dilute a linear monotonic correlation with SIRI while still allowing the highest SIRI tertile to distinguish patients with more advanced vascular pathology. Furthermore, SIRI incorporates monocytes and reflects acute innate immune activation, which may be more tightly coupled to the ischemic/lacunar pattern emphasized by the Staals scale than the hemorrhage-weighted pattern captured by the modified Rothwell scale. This scale-dependent discrepancy underscores the importance of considering scoring methodology when interpreting inflammatory marker associations in CSVD research and argues for harmonized scoring approaches in future multi-center studies (see Figure 4).

Although the ROC analyses indicated that SII and SIRI could discriminate moderate–severe CSVD with statistical significance, the AUC values (0.615–0.635) were modest, indicating that these inflammatory indices alone are insufficient as standalone clinical predictors. This is not unexpected, because CSVD pathogenesis involves multiple pathways—including hypertension, hypoperfusion, oxidative stress, and genetic susceptibility—beyond inflammation alone (Markus and de Leeuw, 2023). In exploratory *post-hoc* analyses, we combined SII or SIRI with age and hypertension; the composite models yielded AUCs of 0.664–0.746, suggesting that a multi-marker approach may provide better risk stratification than inflammatory indices alone. However, these results are *post-hoc* and should be validated in independent cohorts before any clinical application can be considered.

CSVD encompasses a spectrum of pathological processes characterized by heterogeneous etiologies that mainly affect the brain’s small arteries, arterioles, venules, and capillaries (Pantoni, 2010). Accumulating evidence suggests that CSVD correlates to age-related neurological deficits, including cognitive impairment and gait disturbances in the elderly population (Wardlaw et al., 2019). In recent years, the relationship between CSVD and PD has garnered increasing attention, with emerging epidemiological studies largely supporting a potential correlation between CSVD and motor/cognitive impairment of PD, albeit with some inconsistent findings (Sanchez et al., 2025; Jacob et al., 2023; Shen et al., 2023; Wan et al., 2022). Cross-sectional investigations have demonstrated that PD patients with more severe CSVD exhibit more serious motor impairments, particularly gait dysfunction (Chen et al., 2020; Ma et al., 2021). A recent meta-analysis further revealed that CSVD burden is associated with multi-domain cognitive impairments of PD (Wan et al., 2022). Moreover, longitudinal cohort studies have extended these findings by implicating CSVD as a potential etiological contributor to PD pathogenesis (Jacob et al., 2023; Mao et al., 2022; Paolini Paoletti et al., 2021). For instance, a large-scale cohort study indicated that baseline CSVD markers, specifically elevated white matter hyperintensity (WMH) volume and increased lacunar infarct counts, are associated with a heightened risk of incident PD (Oveisgharan et al., 2021). These results align with neuropathological evidence demonstrating that baseline WMH severity and postmortem cerebrovascular pathology correlate with accelerated progression of PD (Buchman et al., 2019). Another longitudinal study exploring CSVD progression and Parkinsonism revealed that both baseline CSVD severity and its dynamic progression independently predict long-term Parkinsonian syndrome trajectories, thereby supporting an association between CSVD progression and parkinsonism (Jacob et al., 2023).

Studies have shown that inflammation plays a significant role in both PD and CSVD, and may serve as a bridge in the interaction between CSVD and PD. In 1988, McGeer and coworkers provided the first neuropathological evidence of neuroinflammation in PD by describing large numbers of reactive microglial cells, including IL-1β, TNF-α and IL-6, etc. in the substantia nigra of patients in postmortem brain samples (Jurcau et al., 2023). Subsequently, more and more evidence emphasizes the role of peripheral inflammation in the occurrence and development of many neurodegenerative diseases, including PD (Muñoz Delgado et al., 2023; Forloni, 2023; Tansey et al., 2022; Pajares et al., 2020).

Similarly, inflammation plays an important role in the pathological mechanism of CSVD. Epidemiological studies have shown that many inflammatory markers, such as CRP, cytokines, and homocysteine might be associated with the occurrence, severity, and progression of CSVD, although the research conclusions are not consistent (Del Cuore et al., 2022). Recently, A systematic review also analyzed and confirmed the correlations between multiple inflammatory markers and CSVD. However, the correlation between different inflammatory markers and different subtypes of CSVD varied, and this correlation might differ among different populations (Low et al., 2019).

Moreover, compared with single blood inflammatory markers, combining multiple markers might enhance the correlation and improve the assessment performance (Wan et al., 2023). In recent years, studies on the correlation between the new inflammatory composite index and CSVD, as well as PD, have gradually increased. A study based on NHANCE indicates that a higher SII can increase the incidence of PD, especially in females (Liu et al., 2025). Another cross-sectional study found that SII is an important factor influencing the motor function status of PD (Li et al., 2021). Recently, a cohort study based on the data of the PPMI cohort has shown that a higher NLR is associated with lower DAT levels in the caudate and putamen nuclei (Muñoz Delgado et al., 2023). Another large cross-sectional study based on the Poly-vasculaR Evaluation for Cognitive Impairment and vascular Events (PRECISE) study indicates that the number of peripheral neutrophils, NLR, and SII are associated with the severity of CSVD in the community population (Jiang et al., 2022).

Nevertheless, the correlation between SII/SIRI and CSVD in PD patients has not been previously reported. Our study provides the first evidence for this association. Several molecular pathways may underlie the observed link between systemic inflammatory indices and CSVD severity in PD. Elevated SII reflects neutrophilia combined with relative lymphopenia, a profile indicative of a pro-inflammatory state. Activated neutrophils release reactive oxygen species via NADPH oxidase, matrix metalloproteinases, and neutrophil extracellular traps, all of which can directly damage cerebral microvascular endothelium and degrade tight-junction proteins such as occludin and ZO-1 (Low et al., 2019; Evans et al., 2021). Concomitant lymphopenia may reflect impaired adaptive immune regulation, further amplifying vascular injury. In the specific context of PD, aggregated *α*-synuclein can act as a damage-associated molecular pattern, activating peripheral monocytes and neutrophils through toll-like receptor 4 and promoting the release of interleukin-6 and tumor necrosis factor-α (Tansey et al., 2022). These circulating cytokines disrupt endothelial nitric oxide signaling and increase blood–brain barrier permeability, facilitating the entry of peripheral immune cells into the perivascular space and exacerbating CSVD pathology (Inoue et al., 2023). The SIRI index additionally incorporates monocytes, which can differentiate into perivascular macrophages and contribute to vessel wall remodeling. Furthermore, recent evidence directly links peripheral inflammatory imbalance to dopaminergic neuron loss: in the PPMI cohort, a higher neutrophil-to-lymphocyte ratio was associated with lower striatal dopamine transporter availability (Muñoz Delgado et al., 2023). Taken together, SII and SIRI may capture a feed-forward loop in which α-synuclein-driven systemic inflammation aggravates cerebral small vessel damage, potentially accelerating both motor and cognitive decline in PD.

Dopaminergic medications have been reported to modulate peripheral immune function. In our cohort, LEDD did not differ significantly between the mild and moderate–severe CSVD groups (Table 1), and additional adjustment for LEDD in sensitivity analyses did not materially alter the associations between SII/SIRI tertiles and moderate–severe CSVD (Supplementary Table 1). This suggests that the observed associations are largely independent of current dopaminergic treatment burden, although subtle immunomodulatory effects of long-term dopaminergic therapy cannot be entirely excluded.

Although our research provided epidemiological evidence for the association between SII as well as SIRI and CSVD in PD patients, some limitations should be considered. First, this is a single-center, cross-sectional study with a modest sample size (n = 167), which cannot establish causality or directionality between SII/SIRI and CSVD in PD. The cross-sectional design precludes determining whether systemic inflammation precedes CSVD progression or vice versa; prospective longitudinal studies with repeated measures of both inflammatory indices and neuroimaging markers are needed. Second, the sample is drawn from one Chinese hospital, which may limit generalizability to other ethnicities or healthcare settings. Third, the absence of a non-PD control group limits our ability to determine whether the observed associations are specific to PD or reflect the general relationship between inflammation and CSVD in aging populations. Fourth, the exclusion of patients with recent infections, autoimmune diseases, or anti-inflammatory/immunosuppressive medication use-while necessary to reduce confounding-may have selected for a relatively healthier PD subgroup, potentially underestimating the true strength of the association. Fifth, despite adjusting for major confounders, residual confounding from unmeasured factors such as physical activity, dietary habits, gut microbiome composition, or subclinical atherosclerosis cannot be excluded. Finally, the modest AUC values underscore that SII and SIRI only partially explain CSVD severity; larger multi-center cohorts with multi-group designs are required to validate these findings and develop more comprehensive risk-stratification models.

## 5 Conclusion

Our research demonstrates that higher levels of SII and SIRI are independently associated with more severe CSVD in patients with PD. However, the discriminative performance of these indices alone is limited. The directionality and causality of the associations remain to be clarified in longitudinal studies.

## Data Availability

The raw data supporting the conclusions of this article will be made available by the authors, without undue reservation.
