# Supplementary material for: Systemic inflammatory index correlates with cerebral small vessel disease in Parkinson’s disease
Source: Front Aging Neurosci. 2026 Jun 3;18:1748084. doi: 10.3389/fnagi.2026.1748084 (PMC13272401; doi:10.3389/fnagi.2026.1748084)
Supplement: Supplementary file 1 [file Table_1.DOCX]

Supplementary Material

# Supplementary Tables

**Supplementary** **Table 1** **Comparison of the longitudinal change of clinical scale scores in different FIB-4 subgroups over 5 years using general linear mixed-effect models**

| **Characteristics** |  | **Model 1** | | **Model 2** | | **Model 3** | |
| --- | --- | --- | --- | --- | --- | --- | --- |
|  |  | **aOR** (95% CI) | ***P* value** | **aOR** (95% CI) | ***P* value** | **aOR** (95% CI) | ***P* value** |
| **CSVD burden score** | SII |  |  |  |  |  |  |
|  | Tertile 1 | Reference |  | Reference |  | Reference |  |
|  | Tertile 2 | 1.296(0.593,2.830) | 0.516 | 1.215(0.528,2.798) | 0.647 | 1.203(0.521,2.777) | 0.664 |
|  | Tertile 3 | 3.017(1.323,6.879) | 0.009 | 2.919(1.191,7.153) | 0.019 | 2.903(1.183,7.125) | 0.020 |
|  | as continuous variable | 1.001(1.000,1.002) | 0.058 | 1.001(1.000-1.001) | 0.099 | 1.001(1.000-1.001) | 0.099 |
|  | SIRI |  |  |  |  |  |  |
|  | Tertile 1 | Reference |  | Reference |  | Reference |  |
|  | Tertile 2 | 1.176(0.536,2.577) | 0.686 | 1.169(0.513,2.668) | 0.710 | 1.169(0.513,2.668) | 0.710 |
|  | Tertile 3 | 3.380(1.458,7.834) | 0.005 | 4.295(1.681,10.973) | 0.002 | 4.295(1.681,10.973) | 0.002 |
|  | as continuous variable | 1.336(0.968,1.845) | 0.078 | 1.362(0.953,1.946) | 0.090 | 1.357(0.947,1.945) | 0.097 |
| **Modified CSVD burden score** | SII |  |  |  |  |  |  |
|  | Tertile 1 | Reference |  | Reference |  | Reference |  |
|  | Tertile 2 | 3.238(1.474-7.111) | 0.003 | 3.221(1.347-7.704) | 0.009 | 3.200(1.336-7.666) | 0.009 |
|  | Tertile 3 | 3.922(1.751-8.788) | 0.001 | 4.196(1.705-10.327) | 0.002 | 4.170(1.693-10.271) | 0.002 |
|  | as continuous variable | 1.001(1.000-1.001) | 0.059 | 1.001(1.000-1.002) | 0.073 | 1.001(1.000-1.002) | 0.078 |
|  | SIRI |  |  |  |  |  |  |
|  | Tertile 1 | Reference |  | Reference |  | Reference |  |
|  | Tertile 2 | 1.410(0.659-3.017) | 0.377 | 1.441(0.632-3.289) | 0.385 | 1.428(0.626-3.260) | 0.398 |
|  | Tertile 3 | 2.817(1.252-6.334) | 0.012 | 3.303(1.326-8.226) | 0.010 | 3.274(1.311-8.175) | 0.011 |
|  | as continuous variable | 1.309(0.959-1.785) | 0.090 | 1.303(0.923-1.839) | 0.132 | 1.296(0.914-1.837) | 0.145 |

**Notes:** The neutrophil and lymphocyte were not included in the multivariable regression analysis because of collinearity.

Model 1: adjusted for gender and age,

Model 2: further adjusted for hypertension, diabetes, hyperlipidemia, coronary heart disease, smoking, drinking, BMI, TC, LDL, FPG based on Model 1.

Model 3: further adjusted for LEDD based on Model 2.

**Abbreviations:** CSVD, cerebral small vessel disease; SII, Systemic immune-inflammation index; SIRI, systemic inflammation response index; aOR: adjusted odds ratio; CI: confidence interval;

**Supplementary Table 2 Receiver operating characteristic (ROC) analyses of SII, SIRI, and their combinations with age and hypertension for discriminating moderate–severe CSVD.**

| **Scale** | **Model** | **AUC** | **95% CI** | **P value** |
| --- | --- | --- | --- | --- |
| **Total CSVD burden (Staals)** |  |  |  |  |
|  | SII | 0.627 | 0.542 - 0.711 | 0.005 |
|  | SIRI | 0.620 | 0.535 - 0.705 | 0.008 |
|  | SII + age + hypertension | 0.689 | 0.619-0.777 | <0.001 |
|  | SIRI + age + hypertension | 0.684 | 0.604-0.764 | <0.001 |
| **Modified CSVD burden (Rothwell)** |  |  |  |  |
|  | SII | 0.635 | 0.549 - 0.722 | 0.003 |
|  | SIRI | 0.615 | 0.530 - 0.702 | 0.010 |
|  | SII + age + hypertension | 0.746 | 0.671-0.821 | <0.001 |
|  | SIRI + age + hypertension | 0.664 | 0.582-0.747 | <0.001 |

**Abbreviations:** CSVD, cerebral small vessel disease; SII, Systemic immune-inflammation index; SIRI, systemic inflammation response index; aOR: adjusted odds ratio; CI: confidence interval; AUC: Area Under the Curve.
